# Phase separation in Cancer: From the Impacts and Mechanisms to Treatment potentials

**DOI:** 10.7150/ijbs.75410

**Published:** 2022-08-01

**Authors:** Qiu Peng, Shiming Tan, Longzheng Xia, Nayiyuan Wu, Linda Oyang, Yanyan Tang, Min Su, Xia Luo, Ying Wang, Xiaowu Sheng, Yujuan Zhou, Qianjin Liao

**Affiliations:** 1 Hunan Key Laboratory of Cancer Metabolism, Hunan Cancer Hospital and the Affiliated Cancer Hospital of Xiangya School of Medicine, Central South University, Changsha, 410013, Hunan, China.; 2 Hunan Key Laboratory of Translational Radiation Oncology, 283 Tongzipo Road, Changsha 410013, Hunan, China.

**Keywords:** phase separation, cancer, biomolecular condensates, tumor-related signaling pathways, virus-associated tumors

## Abstract

Cancer is a public health problem of great concern, and it is also one of the main causes of death in the world. Cancer is a disease characterized by dysregulation of diverse cellular processes, including avoiding growth inhibitory factors, avoiding immune damage and promoting metastasis, etc. However, the precise mechanism of tumorigenesis and tumor progression still needs to be further elucidated. Formations of liquid-liquid phase separation (LLPS) condensates are a common strategy for cells to achieve diverse functions, such as chromatin organization, signal transduction, DNA repair and transcriptional regulation, etc. The biomolecular aggregates formed by LLPS are mainly driven by multivalent weak interactions mediated by intrinsic disordered regions (IDRs) in proteins. In recent years, aberrant phase separations and transition have been reported to be related to the process of various diseases, such as neurodegenerative diseases and cancer. Herein, we discussed recent findings that phase separation regulates tumor-related signaling pathways and thus contributes to tumor progression. We also reviewed some tumor virus-associated proteins to regulate the development of virus-associated tumors via phase separation. Finally, we discussed some possible strategies for treating tumors by targeting phase separation.

## Introduction

Cancer is a complex disease that is based on several “cancer hallmarks” described by Hanahan and Weinberg in 2011. This includes self-sufficiency in growth signals, genomic instability and mutation, resistance to cell death and avoidance of immune surveillance, and others 1-3. Tumor progression is mainly driven by tumor suppressor gene inactivation and oncogene over activation, such as P53, MYC, RAS, EGFR, etc. 4-8. Some tumor-associated signaling pathways are evolutionarily conserved in mammals, which plays a key role in tumor cell development and differentiation. Increasing evidence has shown that aberrant activation of tumor-associated signaling pathways in many different tumors can induce tumor cells proliferation, metastasis and epithelial-mesenchymal transition, such as Wnt/beta-catenin signaling, Hippo signaling and mTOR signaling 9-11.

In addition, tumor associated virus is also one of the important causes of cancer, which is related to about 20% of human tumors. These tumor viruses can affect various cell activities and lead to the occurrence of human malignant tumors 12, 13. At present, several recognized human tumor viruses have been involved in the development of human cancer, including hepatitis B virus (HBV) and hepatitis C virus (HCV) in liver cancer, EB virus (EBV) in lymphoma, human papillomavirus (HPV) in cervical cancer, Kaposi sarcoma herpesvirus (KSHV) in Kaposi sarcoma 14. Although great progress has been made in identifying driving mutations and related oncogenic signaling pathways of oncogenes or tumor suppressor genes, the exact pathological mechanism of tumorigenesis or tumor development is still largely unknown.

Phase separation is a relatively unfamiliar concept in biology, but it is a very common phenomenon in the field of physical chemistry. It describes the dynamic concentration of biomolecules from a homogeneous environment into a relatively dense phase to form a sparse phase and a dense phase 15. The formation of cellular compartmentalization and membrane-less organelles in cells can be explained by LLPS theory. LLPS occurs when multivalent biopolymers interact instantaneously to coalesce into a dense membrane-less condensate 16-18. The characteristics of LLPS include the liquid properties of the formed condensate droplets, such as spherical, fusion and fission, and then relaxation into a sphere 19. More and more evidences show that LLPS is the basis for the formation of various subcellular membrane-less compartments, such as stress granules(SGs) 20-22, Cajal bodies^23^, nucleolus 24, 25, splicing speckles 26-28, and processing bodies (P-bodies) 29. The proteins involved in the formation of LLPS aggregates usually have intrinsically disordered regions (IDRs). These IDRs may mediate weak-affinity and non-specifically interactions of multiple targets to trigger LLPS 30-32. Many functions of IDR depend on their structural properties, such as spacers, flexible linkers or entropic springs 33, 34.

Growing evidence suggests that LLPS condensates are related to the pathogenesis of neurodegenerative diseases 35. In the past few years, LLPS condensates in cells have been known to be associated with several proteins that accumulate in neurodegeneration, including FUS 36, 37, TDP-43 38, 39, HNRNPA1 40, and DDX 41, as well as Tau 42, 43. Some evidence suggested that there is a closely link between the development of cancer and the formation of phase separation condensate 44-48, such as transcriptional condensates, PRC1 condensates, super enhancers, DNA repair condensates, stress granules, Paraspeckles, SPOP/DAXX bodies and PML foci^49^-^54^. Here, we summarize the latest findings on the function and mechanism of LLPS-related condensates. We discuss recent results that biomolecular condensate is involved in regulating tumor-related signaling pathways, thereby contributing to cancer cell survival. We also summarize the function and mechanisms of LLPS condensates in virus-associated proteins to promote the progression of virus-associated tumors. Finally, we discuss how this LLPS condensate affects cancer treatments.

## Characteristics of LLPS condensates

LLPS is a thermodynamic process that divides the mixture into dense phase and dilute phase to achieve the lowest free energy state 19, 55, 56. Phase separation is the characteristics of many macromolecules, such as proteins, RNA/DNA or their complexes like chromatin 57, 58. LLPS is involved in the assembly process of many membrane-less condensates (Table 1), such as Cajal bodies, nucleoli and nuclear bodies, which is conducive to the efficient or orderly regulation of various complex biochemical reactions in cells 59-62. The weak intramolecular and intermolecular interactions, including electrostatic, cation-pi and pi-pi interactions, hydrophobic, are the driving forces for the formation of LLPS membrane-less organelles 31, 63, 64. The assembly of biomolecular aggregates can be promoted by these weak interactions. Special interactions between the condensates can promote the formation of different compartments 19, 65.

The governing mechanism of phase separation condensates in the cell is a multivalent interaction 66-68. Intrinsic disordered regions (IDRs) and low complexity regions (LCRs) can promote the multivalent interaction of proteins 69. LCRs lack a stable three-dimensional structure, and often serve as a scaffold that interacts with short and flexible interacting motifs 30, 70. Some specific amino acids are often highly enriched in IDRs, such as hydrophilic residues (serine, arginine, glutamine, glutamate, and lysine), aromatic residues (tyrosine, phenylalanine, and tryptophan) and charged residues. These residues contribute to form electrostatic interactions, pi-pi interaction and cation-pi interactions, respectively. In contrast, aliphatic residues are less frequently observed in low-complexity domains, such as leucine, valine and isoleucine 71-73.

Proteins and nucleic acids (DNA and RNA) are the main components and mediators of LLPS. Their biophysical properties and phase separation behaviors can vary them to form a highly multi-component system in condensates 74, 75. Many RNA-binding proteins (RBPs) have IDRs and LCRs, referred to as prion-like domains (PLDs), so that phase separation condensates can be formed in an overcrowded nuclear environment 37, 76-78. The protein containing PLDs is initially concerned because they can be assembled into a self-template protein aggregate. These LLPS aggregates may be infectious because they can spread between individuals 46, 79. Approximately 70 human RBPs contain a PLDs via some database of LLPS-related proteins (Table 2), including TDP-43 (transactivation response element DNA-binding protein 43), FUS (fused in sarcoma), EWSR1 (Ewing sarcoma breakpoint region 1), TAF15 (TATA-binding protein-associated factor 15) and hnRNPA1/A2 (heterogeneous nuclear ribo-nucleoproteins A1/A2) 80. Indeed, Wang et al. predicted the saturation concentration of a class of proteins with domain similar length to PLD and RBD of FUS family proteins by using a specific model, and identified some proteins that may provide key scaffold functions for many biochemical compartments in cells 76. Other proteins containing repetitive sequences of Src homology 3 (SH3) domain and proline-rich motifs (PRMs) can also be used as scaffold proteins. Phase separation can be driven by these multivalent SH3/ RPM domains in a concentration dependent manner 67, 74.

Biological macromolecular phase separation is not limited to proteins. RNA is another important component of phase separation condensates. In fact, RNA is widely involved in the formation of RNA/protein-rich membrane-less aggregates in cells by promoting the LLPS 44, 81. For example, the two cytoplasmic RNA particles, SG and P body driven via LLPS are responsible for different main functions, but they also exchange mRNA in each other and share many RBPs 82, 83. Additionally, RNA is an ideal scaffold element for its single-stranded, multivalent, and flexible structures. For example, long non-coding RNAs (lncRNAs) participate in the formation of membrane-less organelles as scaffolds, such as NEAT1 and HSATIII binding for many specific proteins in nuclear body, and keep the dynamic shuttle of proteins and RNAs in nucleoplasm.^49^, ^84^. The formation of phase separation is closely related to the type and concentration of RNA. High RNA/protein ratio can inhibit the formation of phase separation droplets, while low RNA/protein ratio can promote the formation of phase separation droplets. The decrease of nuclear RNA level or genetic alteration of RNA binding leads to excessive phase separation in cells, which promotes the formation of cytotoxic solid like aggregates 37.

## The regulation of LLPS condensates

The phase separation of the protein is strictly controlled by various mechanisms. The multivalent affinity of intermolecular and intramolecular can be regulated by physical conditions such as pH, temperature, ion concentration and osmotic pressure, thereby changing the phase separation behaviors of the biomolecular system 19, 44, 85. Recent results suggest that ParB phase separation condensate needs the ATPase activity of para to maintain. Further experiments show that motor protein can participate in the control of LLPS droplet number and subcellular localization 86. Interestingly, the size, number and subcellular localization of some nucleolar compartments, such as stress granules, heterochromatin domain or P granules, can be controlled by ATP by the similar mechanisms, and after ATP deletion, the fluidity of stress granules was significantly inhibited^61^, ^87^, ^88^.

**Figure 1:**
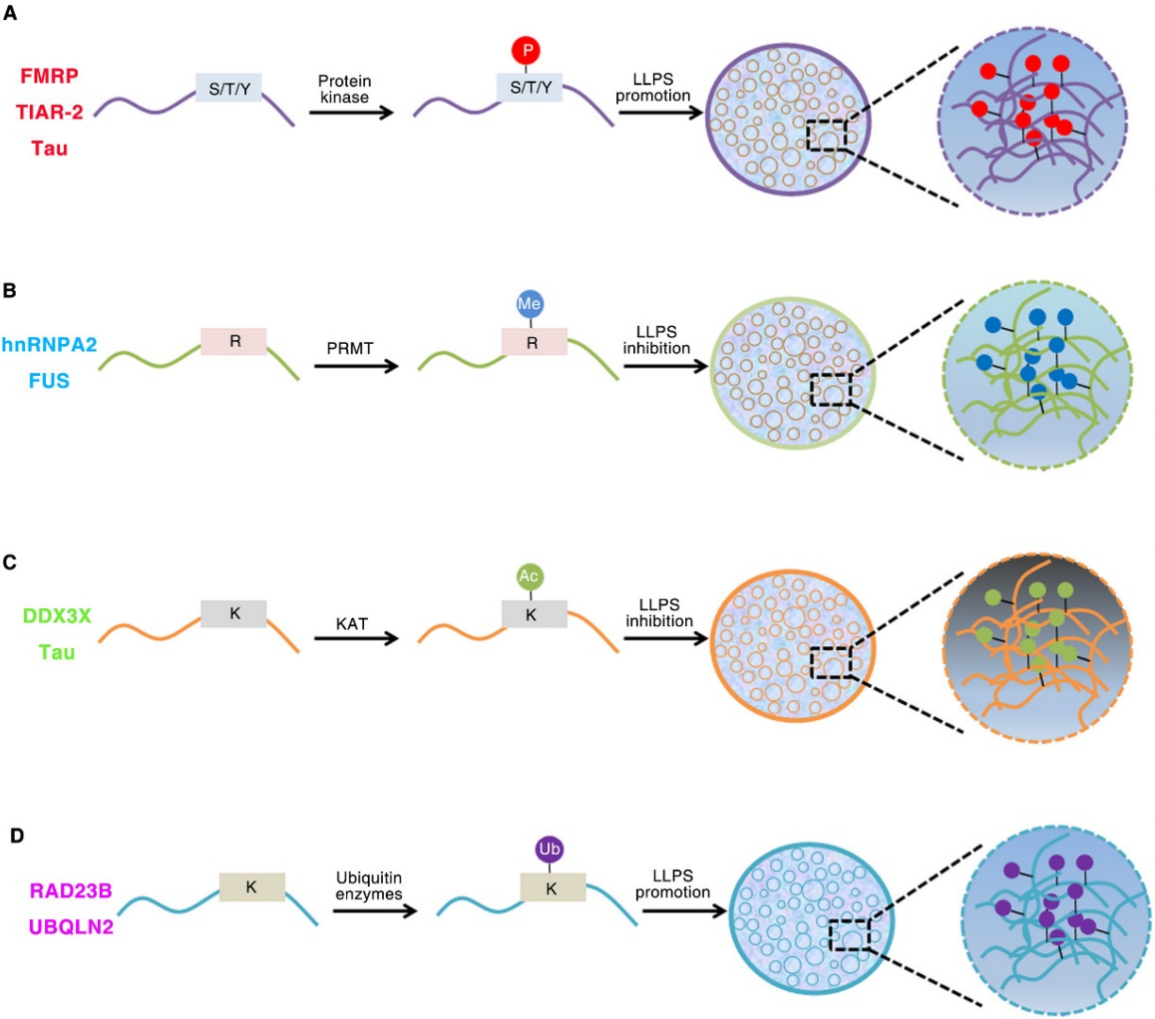
** Role of Post-translational modifications (PTMs) of intrinsically disordered proteins regulates LLPS.** (A). Phosphorylation of FMRP, TIAR-2 and Tau changes the intermolecular interactions and thus promotes FUS/RNA phase separation. (B). Methylation of hnRNPA2 and FUS inhibits the phase separation by weakening the cation-π interactions. (C). DDX3X and Tau IDRs acetylated by lysine acetyltransferase results in impaired phase separation. (D). The RAD23B and UBQLN2 formed LLPS by triggering the multivalent interactions between ubiquitin-associated domains and ubiquitin chains of ubiquitinated proteins.

IDRs are enriched in post-translational modifications (PTMs) sites. These modifications can result in changes in secondary or tertiary structure and can create or destroy interaction sites 32. PTMs can have a strong effect on the charge state and/or binding motifs of proteins, and thus are primary regulators of LLPS (Figure 1). Phosphorylation, acetylation, methylation, sumoylation and ubiquitination are the most common PTMs 58, 89-92. For example, both phosphorylation and phosphomimetic variants in low complexity domain inhibit its prion like characteristics and aggregation tendency. The aggregation tendency of FUS can be significantly reduced when the phase separation of FUS is destroyed by the presence of RNA or salt 93. The occurrence of DDX4 phase separation is driven by its N-terminal RGG-rich domain in vitro, and it can also form liquid condensates in cells. The cation-π interactions between repeated FG and RG motifs can promote the formation of DDX4 LLPS droplets. However, PRMT1 expression can inhibit the formation of DDX4 droplets, mainly because PRMT1 mediates the asymmetric dimethylation of DDX4 41, 94. The phase separation of DDX3X can be driven by the N-terminal IDRs, and the formation of DDX3X droplets can be destroyed by acetylation of multiple lysine residues. HDAC6 can enhance DDX3X phase separation by deacetylating IDRs, which is also necessary to promote SG maturation 95. The IDR of *C. elegans* PcG protein SOP-2 can mediate phase separation, which can be regulated by sumoylation 91. The p62 can form phase separation droplets with liquid properties in vivo which can be induced by adding k63 ubiquitin chain, so that a large number of ubiquitin signals are enriched in p62 droplets 90.

**Figure 2:**
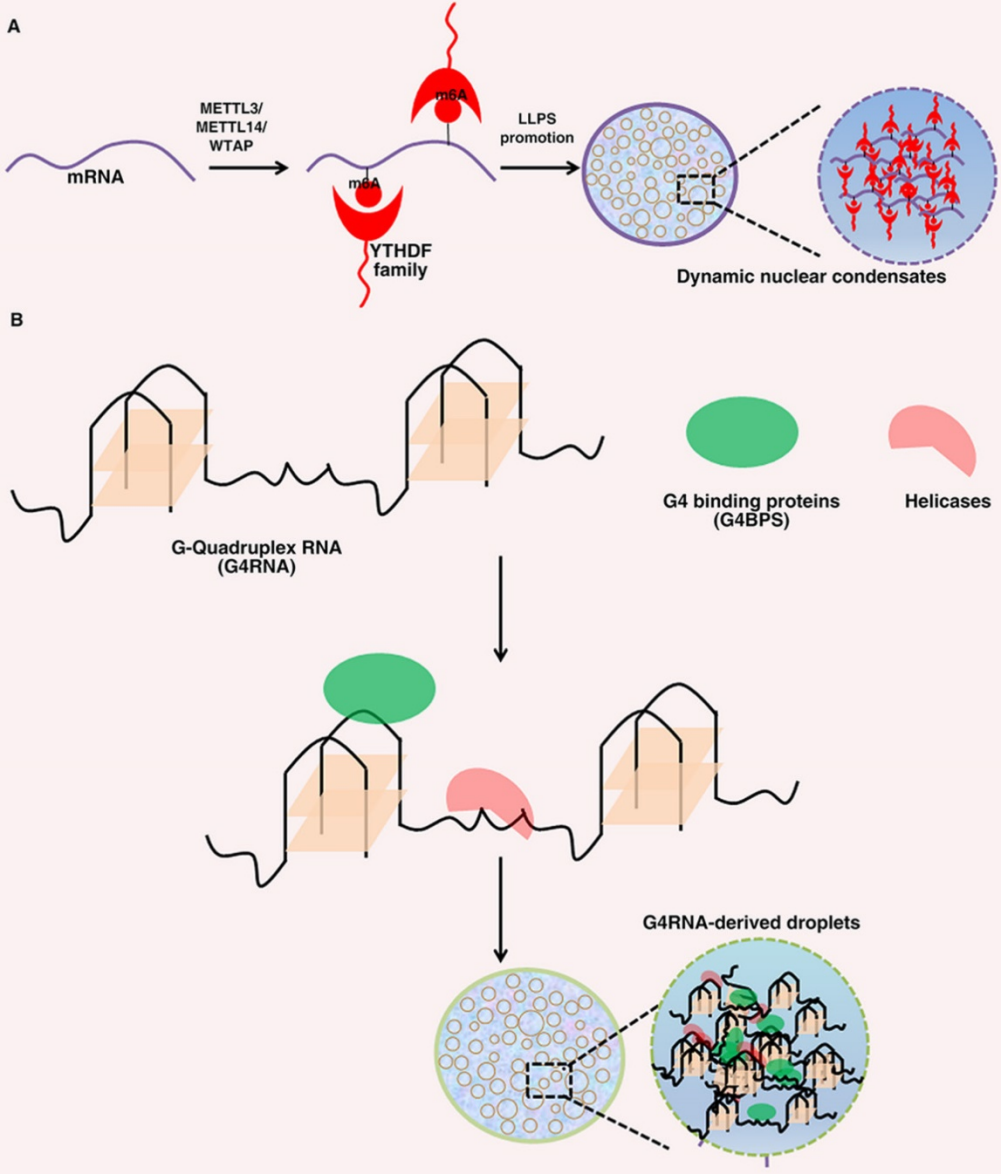
**Roles of N6-methyladenosine(m6A) and G-quadruplex Structures of RNA in LLPS.** (A). The METTL3/ METTL14/WTAP writer complex co-transcriptionally methylates mRNAs. A set of YTHDF family ''reader'' proteins bind directly or indirectly to m6A-mRNAs and thus promotes YTHDF-m6A-mRNAs phase separation. (B). As a scaffold, G4RNAs can interact with G4BP and RNA helicase to promote phase separation.

LLPS has been found to be regulated by post transcriptional modifications (Figure 2) 74. RNA N6-methyladenosine (m6A), as the most common RNA modification, has been reported to be related to the progression of a variety of life activities and diseases 96. YTHDF1-3 is a cytoplasmic m6A binding protein, which has been proved to undergo phase separation both in cells and in vitro. YTHDF proteins can bind to methylated mRNA, resulting in phase separation 97. Similarly, Wang, et al. also reported that m6A enhanced the phase separation ability of YTHDF2 through experiments in vitro and in vivo. In cells, YTHDF2 itself has a weak ability to undergo LLPS, and its phase separation ability is significantly enhanced after binding with m6A mRNA. Although YTHDF2 protein itself can form phase separation droplets in vitro, the addition of m6A modified RNA significantly promotes its LLPS ability 98. Recent studies have reported that highly active enhancer RNA (eRNA) can be modified by m6A to recruit YTHDC1 to form a LLPS condensate, which depends on its C-terminal disordered region and arginine residues. The formation of BRD4 coactivator LLPS condensates can be promoted by YTHDC1/m6A-eRNA phase separation condensate co-mixes 99. In addition, mRNA degradation can be promoted by YTHDF1 phase separation and the interaction of YTHDF1-AGO2 100. These studies showed that the composition of intracellular LLPS transcriptome can be regulated by the distribution and number of m6A sites in mRNA, which indicates that phase separation can control the cellular characteristics of m6A modified mRNA.

RNA G-quadruplex is a secondary structure of nucleic acids formed in guanine rich sequences. It can interact multivalent with RNA binding proteins and RNA, which also makes it a favorable scaffold for RNA-driven phase separation (Figure 2) 84. SHR mRNA has RNA G-quadruplex structure, which can undergo LLPS under physiological conditions. Under the condition of more G-quadruplex, the ability of G-quadruplex to trigger phase separation will be significantly enhanced. In addition, the formation of phase separation is closely related to the number of G-quadruplex and the length of loops 101. Recently, Liu, et al. reported that single stranded DNA with parallel G-quadruplex structure can functionally cooperate with G-quadruplex binding protein to form phase separation droplets by using specific giant membrane vesicles as a protocell model 102. In addition, the LLPS of FUS condensate formation is significantly enhanced through the interaction between FUS and G-quadruplex-RNA 103. These studies have indicated that special RNA secondary structures may have an important role in forming different phase separation condensates in a cellular environment.

## The role of LLPS in oncogenic signaling

**Figure 3:**
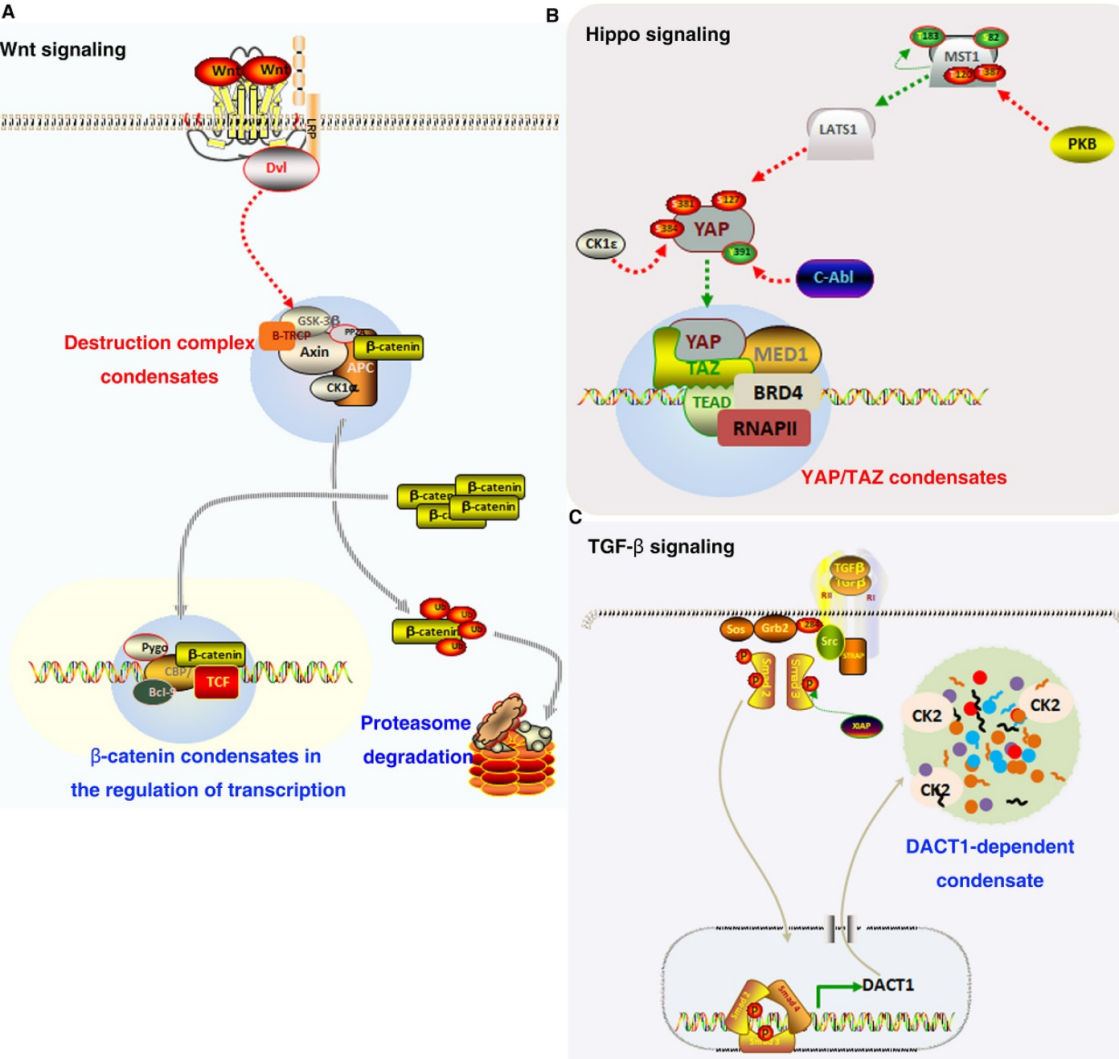
**Role of LLPS in oncogenic signaling.** (A). In Wnt/β-catenin signaling pathway, Axin, and APC assemble into a destruction complex condensate that recruits other members such as GSK3 and CKI. β-catenin accumulates and is transported to the nucleus, where it may localize to condensates at super-enhancers to activate the expression of target genes. (B). YAP/TAZ condensates co-localize with TEAD and recruit RNA Pol II to promote the expression of downstream target gene. (C). TGF-β promotes the expression of DACT1, which through LLPS is required for compartmentalising hundreds of proteins including CK2.

Since “phase separation” is involved in various life activities of cells, its abnormal state will inevitably lead to the occurrence of many diseases. Neurodegenerative diseases including frontotemporal dementia (FTD) and amyotrophic lateral sclerosis (ALS) have been linked to disruption of the components and properties of LLPS condensates 104, 105. The relationship between the LLPS stress particles and these ALS/FTD-associated proteins, including HNRNPA1/HNRNPA2, TDP-43 and FUS, has always been the main focus to correlate LLPS particles to neurodegeneration 106. More and more evidences show that LLPS are involved in many major cellular processes, such as heterochromatin and genome organization, transcription, and stress responses 58, 107-110. Some studies have reported that LLPS can also occur on many carcinogenic signaling molecules 111, 112. Here, we will elucidate the relationship between oncogenic signaling pathways and mis-regulated phase separation (Figure 3). Additionally, we also discuss the mechanisms of phase separation of oncogenic signaling molecules and its potential significance.

### LLPS in p53 signaling

The p53 protein and its cellular pathway are highly conserved in evolution, which can lead to cell death, mediate tumor inhibition or maintain cell homeostasis through a group of regulatory, informed and comprehensive responses to environmental disturbances 113. p53 can act as an internal monitor of many cellular stresses and DNA damage response, such as telomere shedding, mitochondrial and ribosomal biological changes, spindle poisoning, starvation, hypoxia or oncogene activation. Depending on the degree of cell damage, p53 can induce cell cycle arrest or cell death, aging and DNA repair 114, 115. In 50% of human cancers, p53 mutations hinder their binding to the specific target sequence. Therefore, many studies have been carried out on the function of p53 and the consequences caused by its loss of function 116.

Recently, another important aspect of p53 that has been reported is that it can play its function by participating in the formation of LLPS liquid-like condensates. For example, p53 is involved in the formation of PML and Cajal bodies under stress response conditions 117, 118. P53 itself has the potential to form phase separation liquid-like condensates, which can be regulated by post-translational modification and other cellular molecules 116. Some studies have found that p53 protein amyloid formation exists in human cancer tissues. The formation of p53 amyloid protein in cells can lead to its functional inactivation and promote its transformation into oncoprotein. Cancer-associated mutation of p53 can accelerate the protein aggregation and amyloid formation by destroying the folding of p53 core domain 116. In addition, the formation of aggregation structure in cancer may be caused by the formation of p53 phase separation condensate, such as mutant amyloid oligomer 119. P53 signal transduction and DNA damage response can be regulated by p53 binding protein 1 (53BP1) 120. 53BP1 can form phase separation droplets, which enrich tumor suppressor protein p53. The expression of p53 target gene and 53BP1-dependent induction of p53 can be inhibited by destroying the phase separation of 53BP1 51. The scaffold protein AHNAK can regulate the phase separation potential of 53BP1 by binding to its oligomeric domain. The excessive accumulation of 53BP1 in chromatin, enhancement of its LLPS, increasing of p53 response, destruction of the survival of cancer cells, and the aging of non-transformed cells are closely related to the loss of AHNAK 121. These studies suggest that one of the reasons for the loss of p53 function may be the phase separation of p53 and the formation of amyloid protein. Lemos, et al. found that the small molecule compound aminothiazole can destroy p53 condensate by interacting with p53, which indicates that the compound changes the condensation behavior of p53 according to the type of p53 mutation. Furthermore, the compound does not cause reactivation of mutant p53 and is active on p53 phase separation condensate. These results provide evidence for p53 phase separation condensation of mutations in cells and provide tools to regulate this process 122.Therefore, targeting the formation of p53 phase separation may become an important way of cancer treatment.

### LLPS in Wnt/β-catenin signaling

Wnt/β-catenin signaling is one of the key pathways controlling stemness and development, and is closely related to cancer 123. Wnt/β-catenin signaling participates in a variety of tumor physiological processes such as proliferation, migration/invasion and apoptosis 124-126. Wnt/β-catenin signaling pathway generally refers to the canonical Wnt signaling, which can be divided into three main components: β-catenin protein, degradation complex and membrane protein three main components 127. The localization of Wnt protein receptors low-density lipoprotein receptor related protein group (LRP5/6) and frizzled (Fzd2) receptors on the cell membrane 128, 129. The Axin, glycogen synthase kinase 3β (Gsk3β), adenomatous colorectal polyps (APC), casein kinase 1α (CK1α), and dishevelled (DVL) protein to form the β-catenin destruction complex. CK1α and GSK3β can promote β-catenin ubiquitination and subsequent proteasome degradation by controlling its phosphorylation successively 130, 131. Un-phosphorylated β-catenin gradually accumulates in cytoplasm and transports to the nucleus to activate Wnt downstream target genes by interacting with lymphoid enhancer-binding factor (LEF) and T cell-specific factor (TCF) co-activators 132.

Recent studies have shown that LLPS can occur in some signaling molecules in Wnt signaling pathway, which is very important for the regulation of Wnt functions 133. IDR in Axin can drive its phase separation and promote the formation of destruction complex. Phase separation phenomenon has also been found in APC molecules, which can enhance the dynamic of Axin phase separation droplets in vitro. The assembly of β-catenin destruction complex and β-catenin phosphorylation by GSK3β/CK1α are driven by phase separation, which then maintain β-catenin protein stability and regulate Wnt/β-catenin signal transduction 133. In vitro, IDRs of APC undergoes phase separation. In colorectal cells, β‑catenin degradation and Axin puncta formation can be promoted by expressing IDR of APC 134. Dvl is a multivalent protein interacting with other Wnt signaling proteins. Dvl was also observed to form puncta in cells, and its DIX domain is important for the puncta formation. Although Dvl protein has been proposed to have an ability to undergo LLPS, there is no experimental evidence that has been reported by far 133, 135, 136. Recent studies have shown that coactivator, mediators and some transcription factors enrich on super enhancers to form phase separation condensates 110, 137. Interestingly, β-catenin interacts with DNA binding factors and selectively occupies the super enhancer to form a phase separated condensate 138. These studies provide the possibility that phase separation is involved in the assembly of Wnt pathway molecules to regulate the development of cancers.

### LLPS in Hippo signaling

The Hippo pathway is a conserved pathway that plays a key role in organ development, immune regulation and tissue regeneration 139. A variety of cancer progression has been found to be related to the dysregulation of Hippo pathway, such as lung, colorectal, ovarian and pancreatic cancers 140-143. The Hippo pathway is composed of a huge protein network, which not only regulates the growth of different tissues in the process of regeneration and development, but also controls the occurrence and development of cancer in pathological state 10. Many of these functions are mediated by transcriptional factors YAP and TAZ, which directly regulates gene expression by controlling the transcription factor TEAD family 139.

Some studies have highlighted that the nuclear cytoplasmic shuttle behavior of Hippo pathway transcription coactivators YAP and TAZ is much more dynamic than previously recognized, and that YAP and TAZ are also regulated by LLPS 144. TAZ forms nuclear condensates through LLPS to compartmentalize BRD4 and coactivators MED1, the transcription elongation factor CDK9 for transcription, and its DNA-binding cofactor TEAD4. Hippo signaling pathway can negatively regulated the phase separated ability of TAZ via LATS-mediated mediated phosphorylation. In addition, the coiled-coil domain can drive the phase separation of TAZ 145. Some super enhancer markers such as Oct4, Sox2, H3K27ac and Nanog can co-locate with YAP in mouse embryonic stem cells. In addition, YAP also guides the formation of Med1 labeled aggregates at its binding site through LLPS 146. Mechanistically, the activation of TAZ in the nucleus and the occurrence of TAZ phase separation are regulated by paraspeckle protein NONO. Overexpression of NONO promoted nuclear TAZ phase separation, while low expression of NONO decreased nuclear TAZ phase separation. Moreover, the low expression of NONO inhibited the interaction of TAZ with enhancers and TEAD 147. In addition, the phase separation of LATS1 can be promoted by phosphatidic acid-binding lncRNA SNHG9, thereby promoting carcinogenic YAP signaling. These findings have revealed a tumor-associated lncRNA as a key regulator of YAP by facilitating the formation of LATS1 phase separation condensates 148. Taken together, it can be assumed that Hippo signaling molecules are activated through phase separation and thus play a key role in the occurrence and progression of tumors.

### LLPS in TGF-β signaling

Transforming growth factor β (TGF-β) signaling pathway is an evolutionarily conserved pathway and plays a key role in some biological processes, such as cell apoptosis, migration, growth, differentiation, tumorigenesis and development 149. TGF-β receptor can be activated by its ligand to promote the phosphorylation of serine/threonine residues, and then induce the phosphorylation of intracellular effector SMADs 150. The activated SMADs Protein can transfer to the nucleus, activate the transcription of its target genes and regulate cellular functions 151. Smad family consists of many proteins whose main function is to transducing extracellular signals to the nucleus, in which SMAD4 is the key regulator BMP and TGF-β signaling pathway, while SMAD2/3 mainly controls TGF-β signal transduction of subfamily members 149.

Recently, some signaling molecules with IDR in TGF signaling pathway are enriched in super-enhancers condensates by phase separation. TGF-β signaling factors SMAD3 can form nuclear foci when the signaling pathway is activated. The results show that these SMAD3 nuclear foci are condensates formed by phase separation 138. In addition, TGF-β signal can induce DACT1 to form phase separated condensates in cytoplasm, which can inhibit the function of Wnt signaling pathway. The deletion of IDR in DACT1 can destroy its ability to form phase separation condensate and inhibit Wnt pathway. Moreover, the role of DACT1 in breast cancer and prostate cancer bone metastasis is also dependent on the maintenance of DACT1 aggregates in cellular 152. These studies suggest that phase separation may influence tumor progression by regulating TGF-β signaling pathway.

### LLPS in AMPK signaling

In eukaryotic cells, AMP-activated protein kinase (AMPK) can monitor ATP: ADP: AMP ratio through as an energy sensor 153. The increase of intracellular ADP/AMP relative level or the decrease of ATP level can activate AMPK, including stress responses triggered by tissue ischemia, hypoxia, muscle resection or glucose deprivation 154. AMPK has been proved to be closely related to the occurrence and progression of tumors, such as lung, liver and pancreatic cancer 155-157. A-kinase anchoring protein 1 (AKAP1) can be phosphorylated by AMPK in mitochondria. As a scaffold protein of protein kinase A (PKA), AKAP1 can promote oxidative phosphorylation and mitochondrial fusion by promoting the phosphorylation of dynamin-related protein 1 (DRP1) and mitochondria fusion factor 153, 158.

Recently, Zhang, et al. reported that RIA, a type I regulatory subunit of PKA, can undergo phase separation and form liquid-like biomolecular aggregates enriched in cAMP and PKA, which is essential for cAMP compartmentation. They further showed that RIA phase separation can be effectively inhibited by PKA fusion oncoprotein related to atypical liver cancer, which can induce abnormal cAMP signaling. The destruction of RIA-LLPs in normal cells can promote cell transformation and induce cell proliferation. Their work suggested phase separated as an important assembler of signaling condensates and highlights the pathological significance of this dynamic structural disorder 111.

### LLPS in mTOR signaling

Mechanistic target of rapamycin (mTOR) is usually assembled into several complexes, such as mTOR complex 1/2 (mTORC1/2). As a protein kinase, mTOR participates in the regulation of cell survival, growth, immunity and metabolism 159. A variety of diseases, including tumors, are related to mTOR signaling deregulation 160. mTORC1 is sensitive to rapamycin and contains mTOR, mLST8 and RAPTOR. mTORC2 is not sensitive to rapamycin and contains mTOR, MAPKAP1, mLST8 and RICTOR^161^. Growth factors and nutrition can activate mTORC1. Different from mTORC1, the activation of mTORC2 only needs growth factor signaling, but its specific molecular mechanism is not completely clear 9. mTORC1 mediates the expression or phosphorylation of eIF4E, S6K1, 4E-BP1, lipin1, ULK1, TFEB, ATF4, HIF1α, etc., and regulates the nucleotide, lipid and protein synthesis, thereby controlling cell proliferation, growth, metabolism and autophagy. mTORC2 controls the phosphorylation of SGK, PKC and Akt, etc., thus regulating cell apoptosis growth, migration and metabolism^9^, ^160^, ^162^.

Recently, Zhang, et al. showed that heat stress can promote mTORC1 mediated PGL-1/3 phosphorylation and LLPS of PGL-1/-3 to form PGL particles resistant to autophagy degradation. Moreover, the accumulation of PGL phase separation particles is an adaptive response to thermal stimulation to maintain embryo survival. They found that mTORC1-regulated phase separation of PGL-1/-3 acts as a switching pressure sensor, coupling LLPS to autophagic degradation and stress adaptation 89. In addition, Schilling, et al. determined that mTOR is a key regulator of survival motor neuron (SMN) phase separation condensation in Cajal body through siRNA-based system. Proteomic analysis revealed that there was TOR dependent phosphorylation in the subunits of SMN complex. They also demonstrated that the ability to condense in Cajal bodies by phase separation can be controlled by phosphorylation of serine 49 and 63 of SMN. Their findings link cellular energy with SMN complex phase separation condensation and UsnRNP biogenesis, and emphasize the regulation of TOR signaling as a reasonable concept for the treatment of SMN-related diseases 163. These results showed that LLPS plays a key role in the mTOR pathway, but further evidence is needed to determine whether phase separation regulates tumor progression by activating the mTOR signaling pathway.

### LLPS in autophagy

Autophagy is an intracellular protective mechanism that can transfer damaged cellular substances to lysosomes for degradation, provide molecular precursors and energy, and allow the basic turnover of cellular components 164. Autophagy dysregulation are associated with a variety of diseases. For example, in cancer, autophagy can both inhibit tumor initiation and promote cancer progression 165. Induction of autophagy can be triggered by several intracellular and extracellular stimuli, such as nutrient starvation and serum starvation, oxidative stress and eliminated proteins aggregates, and inhibitors of TOR, e.g., rapamycin 166. The autophagy process consists of four key steps: initiation, nucleation, maturation and degradation, each of which involves many key proteins. Such as, the autophagy related gene 13 (ATG13), ATG101 Unc-51-like kinase 1 (ULK1) and FIP200 play a role in the initiation step. VPS15, Beclin-1, autophagy and beclin 1 regulator 1 (AMBRA-1), ATG14L and VPS34 are involved in the nucleation step. ATG7, ATG10, ATG5, ATG12, ATG3, LC3 (ATG8), lipid phosphatidylethanolamine (PE) 14, phosphoinositol 3-phosphate and ATG4 are involved in the maturation step. SQSTM1 (p62), neighbor of BRCA1 (NBR1), multiprotein HOPS complex, syntaxin 17 and EPG5 are involved in the degradation step 167.

**Figure 4:**
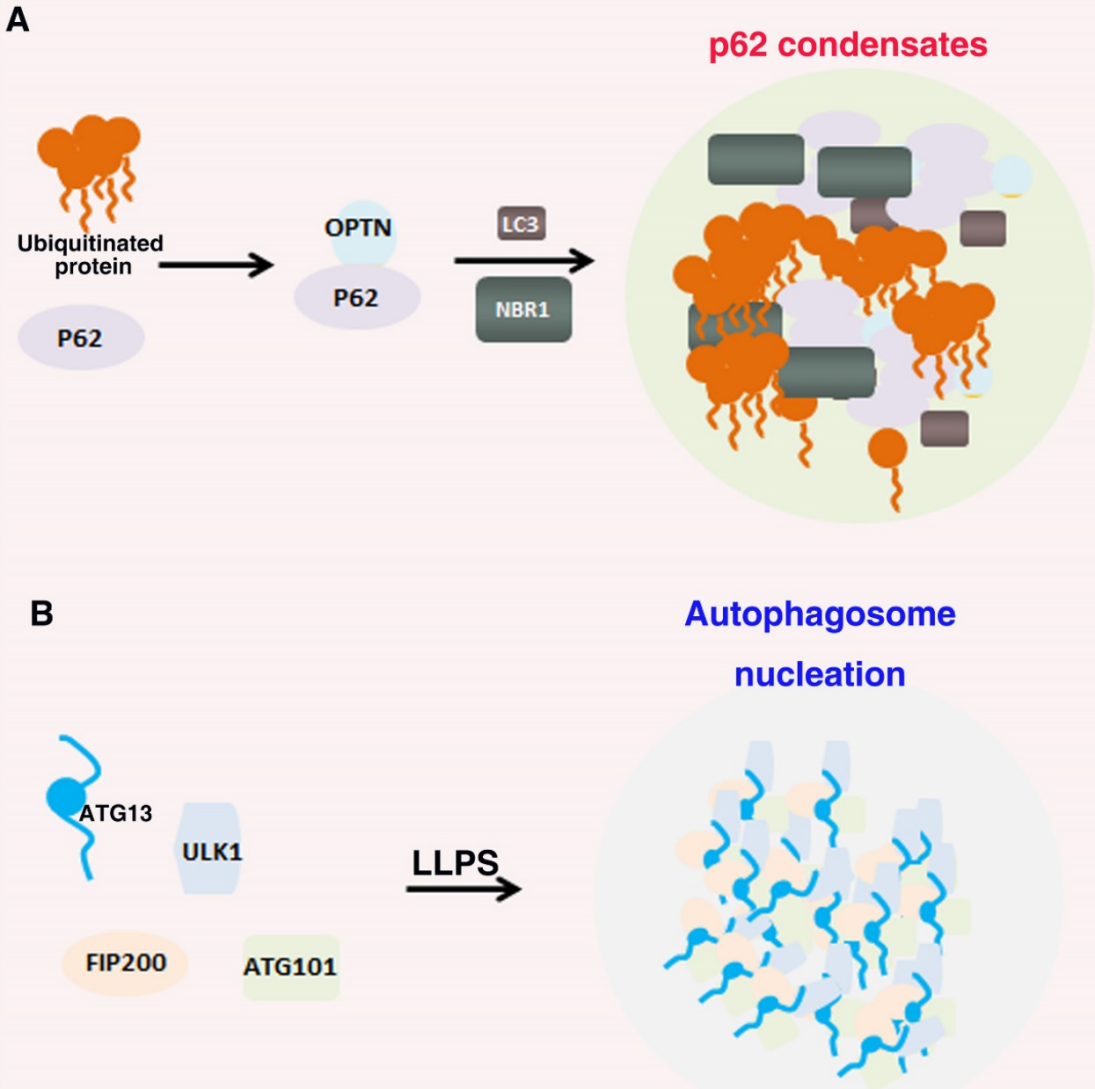
**Role of LLPS in autophagy.** (A). p62 interaction with NBR1 proteins and bind to ubiquitin and the polyubiquitin chains of autophagy receptor OPTN to form autophagy receptor condensates; (B). ULK1 complex contains FIP200, ATG13, ULK1 and ATG101. ATG13 interact with FIP200 by the IDR domains multivalent interactions and thus drive LLPS of the ULK1 complex to recruit downstream autophagy proteins for autophagosome formation.

Many biomolecules of autophagy undergo LLPS that regulate many cellular functions (Figure 4)^168^. P62 can form phase separation droplets with liquid properties, such as fusion and fission, micron sized spheres, and recovery rapidly after photobleaching in vivo. In the mechanism, the interaction ubiquitin with p62, p62 polymerization and the multivalent state of ubiquitin chain can significantly promote the LLPS of p62. Furthermore, post translational modifications such as phosphorylation can also regulate the phase separation of p62 90, 169. In addition, Agudo-Canalejo, et al. also examined how phase separation condensates containing p62 protein in cellular were isolated by autophagosomes, and proved that autophagosome-like vesicles were formed on the surface of protein-free droplets by partial wetting in vitro 170. The preautophagosome structure (PAS) is a LLPS condensates containing Atg proteins. The Atg1 complex initiated by autophagy forms droplets through LLPS, and phosphorylation or point mutation can inhibit LLPS and further destroy the formation of PAS 171. IPMK dysregulation can enhance autophagy activity by activating TFEB, which can form a condensate driven by phase separation with dynamic characteristics. Nuclear TFEB phase separation condensates can participate in the transcription of downstream target genes by interacting with transcriptional condensate MED1 172. Wilfling, et al, reported that a selective autophagy pathway is a LLPS condensates formed by endocytic proteins. They found that endocytic protein Ede1 binds Atg8 and mediates LLPS into condensates 173. These results suggest that the LLPS play a critical, active role in autophagy signaling pathway.

### LLPS in immunity

**Figure 5:**
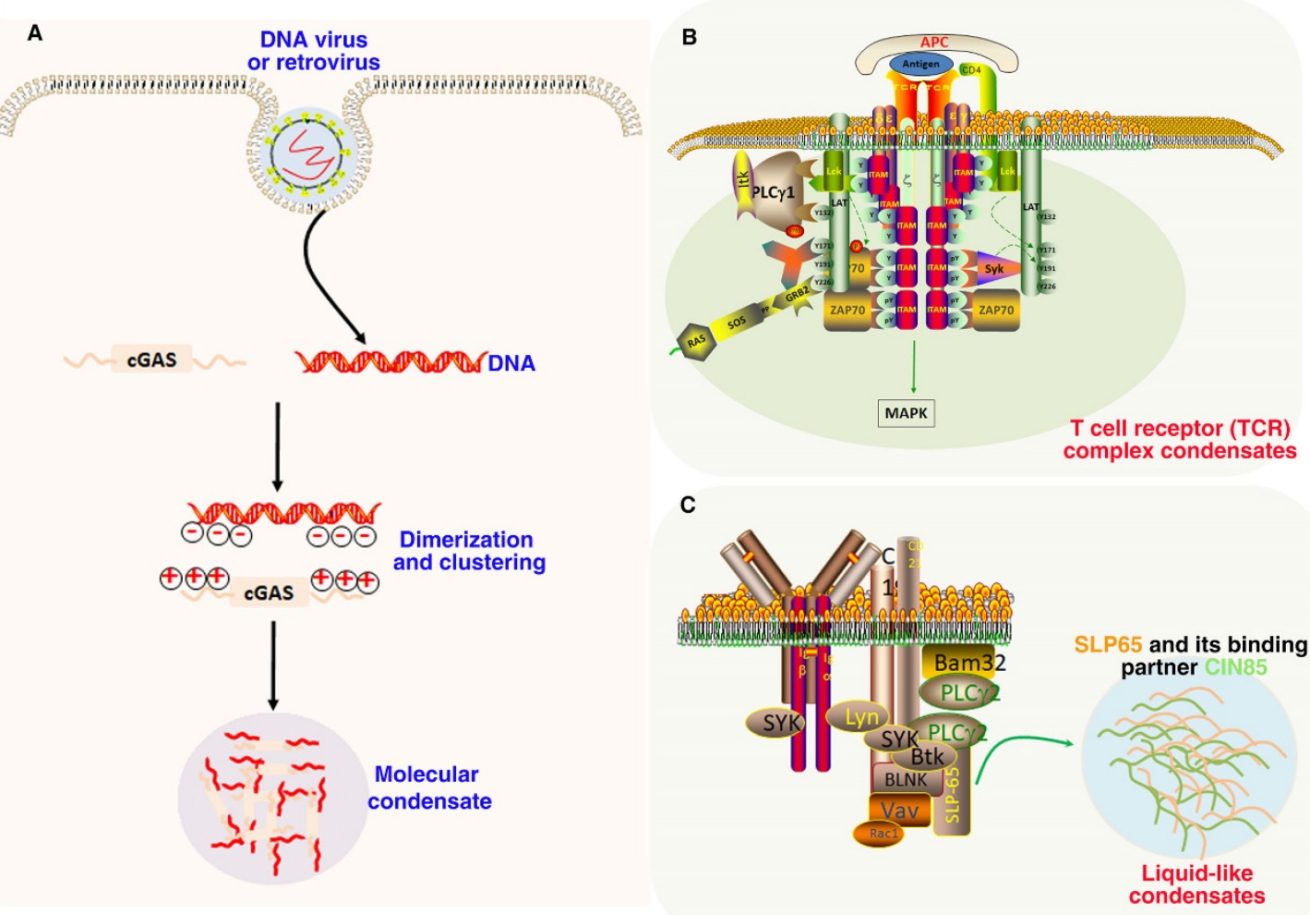
**Role of LLPS in immune signaling.** (A). Double-stranded DNA binding with cGAS prominently promoted their phase separation and the formation of condensates. In these condensates, cGAS is highly concentrated, which further promotes its catalytic activity by changing the multivalence interaction between cGAS and DNA. (B). The TCR complex is phosphorylated by LCK on ITAM domain, which further recruits the kinase ZAP70. The transmembrane protein LAT is then phosphorylated by ZAP70 and drives LLPS through multivalent interactions with GRB2 and SOS1 for MAPK signaling. (C). SLP65 and its binding partner CIN85 form LLPS condensates in the cytosol of B cells through multivalent interactions between the SLP65 and CIN85.

In recent years, a large number of studies are looking for the mechanism of tumor immunity. More and more drugs for immune checkpoint therapy have been applied in a variety of cancers 174. Cancer immune therapy has shown remarkable benefits in the treatment of many tumors 175. The innate and adaptive immune system are closely related to tumor development and induction of anti-tumor immune responses 176. Recently, LLPS has played an increasingly important role in immunology, which also provides a new direction for a deeper understanding of immune response (Figure 5)^15^, ^177^.

The main function of cGAS-STING signaling pathway is to monitor exogenous DNA and activate innate immune response, such as interferon response after pathogen infection. It is mainly composed of the cyclic GMP-AMP receptor stimulator of interferon genes (STING) and the second messenger cyclic GMP-AMP (cGAS) 178. The antitumor immune response can be significantly promoted by the activation of cGAS-STING signaling pathway to produce type I interferon 179. The interaction between DNA and cGAS will strongly induce the formation of phase separation droplets. The LLPS of cGAS-DNA is driven by the increasing of DNA binding valence at the N-terminal of positively charged and disordered cGAS. These findings suggest that DNA promotes the phase separation of cGAS and activates innate immune signaling 180. Moreover, LLPS of cGAS can not only enhance cytoplasmic DNA sensing, but also inhibit TREX1-regulated DNA degradation. The results revealed a new molecular mechanism, that is, cGAS-DNA activates innate immune response and balances cytoplasmic DNA degradation by phase separation 181. Mutant tumor suppressor can regulate cGAS-STING pathway by phase separation, and reveal the function and pathogenesis of NF2-associated tumors by controlling antitumor immunity 182. In addition, in DNA virus infected cells, STING can undergo phase separation to form a biological condensate with an organized membrane structure. The 2'3'-cGAMP can induce the formation of STING phase separation, so as to separate STING-TBK1 from IRF3 and prevent excessive activation of innate immunity 183.

The role of phase separation has also been reported in B cell receptor (BCR) and T cell receptor (TCR) pathways 15. Once TCR is activated, downstream signaling proteins spontaneously aggregate into phase separated clusters, thereby promoting signal output in Jurkat T cells and in vitro. These results suggest that phase separation is closely related to the reconstruction of T cell signal pathway and the promotion of specific biochemical and signal transduction reactions 112. In the study of Huang, et al., authors established a foundation for the dynamic proofreading of receptor-mediated Ras activation. They further demonstrated that this kinetic proofreading was modulated by the LAT (linker for activation of T cells)-Grb2-SOS phosphotyrosine-driven phase transition at the membrane 184. In BCR pathways, cluster BCR is located in the ordered phase-like region, which can sort the key regulatory factors of BCR activation, and a minimum prediction model is proposed, in which cluster receptors stably expand the ordered domain by using super-resolution microscope, resulting in their collective activation 185. In addition, Effective B cell activation requires the LLPS of CIN85, SLP65 and lipid vesicles into phase separation through the vesicle binding of SLP65 and the hybrid interaction between the proline rich motifs (PRMs) of SLP65 and the SH3 domains of CIN85. The results suggested that LLPS, driven by the transient interaction between vesicle and scaffold protein, is a cellular mechanism of organize signal transducers and aggregation 186. In consideration of the important role of LLPS in immune signal activation, it may also provide a new perspective for immunotherapy of tumors.

### LLPS in metabolism

Metabolic reprogramming can be widely observed in the occurrence and development of tumors, which endows tumor cells with the ability of malignant proliferation. Even under aerobic conditions, tumor cells like to perform glycolysis to obtain energy and metabolites. This phenomenon was first discovered by Otto Warburg and is now known as “Warburg effect” or “aerobic glycolysis” 187. Rapid proliferation of cancer cells has been shown to require the support of metabolic reprogramming. The increase of metabolites produced by aerobic glycolysis, such as lactic acid, is related to promoting cancer cell proliferation and metastasis. Therefore, increasing efforts have been invested in trying to develop new therapeutic drugs that target cancer metabolism 188. Glycolysis plays a central role in metabolism, providing energy as well as carbon feedstocks for anabolic pathways Glycolysis can provide energy and carbon materials for anabolic pathway, and play a core role in metabolism. Recently, under specific stress conditions, some glycolytic enzymes were found to be involved in the formation of phase separation condensates 189. The glycolytic bodies formed by LLPS are a new RNP particle. The RNA substrates of the glycolytic bodies reside in glycolysis machinery, and the RNA plays key roles in glycolytic body biogenesis and maintenance 190. Glucose consumption usually increases in cancer cells to support cancer cell proliferation. It was found that Saccharomyces cerevisiae also had “glycolysis” or “G body”. In hypoxia environment, Saccharomyces cerevisiae can concentrate glycolytic enzymes to form a non-membrane bound particle, namely “G body”. Snf1p, a homolog of AMP-activated protein kinase, is necessary for G body formation. The formation of G-bodies can affect cell division by affecting the level of glycolysis 191. A recent study showed that the accumulated glycogen undergoes LLPS, leading to the assembly of Laforin-Mst1/2 complex, thereby isolating Hippo kinase Mst1/2 in glycogen condensates to reduce its inhibition of YAP. Moreover, deficiency of G6PC or PYGL, glycogenolysis enzymes in both human and mice results in glycogen storage diseases along with liver enlargement and tumorigenesis in a YAP-dependent manner. In addition, in humans and mice, the loss of function of PYGL or G6PC, glycogenolysis enzymes will lead to glycogen storage impairment, resulting in the promotion of tumorigenesis in a YAP-dependent manner 192. These studies suggest that metabolism can regulate tumor progression through phase separation.

## LLPS in tumor virus-associated proteins

**Figure 6:**
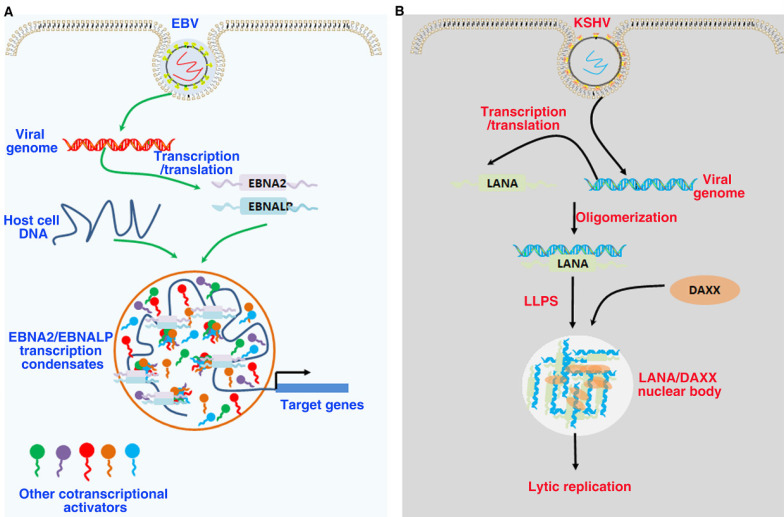
**Role of LLPS in tumor virus-associated proteins.** (A). EBNA2 and EBNALP recruit other coactivators and transcription factors forming phase-separated condensates at enhancer sites to drive gene activation. These are driven in part by the interactions of IDRs. (B). LANA-associated nuclear bodies structures self-assembly through LLPS to build dynamic structures. DAXX is a component of the latent phase LANA-associated nuclear bodies, and the low complexity, multivalent N-terminal domain interactions driving LLPS.

Oncogenic viruses can affect various cellular events and lead to the occurrence of human malignant tumors, which is also one of the most important reasons for the occurrence and development of tumors 12. Approximately 20% of all human oncogenesis is caused by cancer-causing viruses. Oncogenic viruses can cause about 20% of human oncogenesis. Viral infection can cause uncontrolled proliferation, chronic inflammation, and the expression of some key regulatory proteins 13. oncogenic viruses, such as HBV, HPV, EBV, HCV, HTLV-1 and KSHV are associated with approximately 10%-18% of human tumor worldwide 12. The viral protein LLPS participates in a series of regulatory steps in the viral replication and lytic cycle (Figure 6). A key function of phase separation driven by viral-encode proteins is the formation of “viral factories” or “viral inclusions”. The formation of LLPS of some viral proteins may not be related to virus replication and assembly, but interferes with the function of the host cell. This interference may depend on the interaction with cellular proteins or changes in host gene transcription, that is, “LLPS mediated host cell function interference” 193-197.

### LLPS in EBV

EBV is the first oncogenic DNA virus found to continuously infect humans and some other primates. As a class of I carcinogens, EBV is associated with a variety of human malignant tumors, such as epithelial and lymphoid tumors, including gastric cancer, nasopharyngeal carcinoma, Burkitt's lymphoma, Hodgkin's lymphoma and extranodal NK/T cell lymphoma^198^. EBV mainly infects the host in the form of latent infection. EBV has three types of latent infection, of which type III expresses all latent infection genes, such as latent membrane proteins LMP1/2, non-coding EBV encoded RNA (EBER1/2), six Epstein-Barr nuclear antigens (EBNA1, EBNA2, EBNA3A, EBNA3B, EBNA3C and EBNA LP), and viral microRNA (miRNA) 199. EBNA2 and its coactivator EBNALP are the earliest expressed transcription factors after EBV infects B cells. The co-expression of EBNALP and EBNA2 can regulate the expression of specific genes to drive quiescent B cells into the cell cycle, so as to promote the growth and transformation of B cells 200. EBNA2 and EBNALP can be enriched on super enhancers such as MYC and RUNX3 to form phase separated condensates. Destroying the phase separation of EBNA2 and EBNALP with 1,6 hexanediol can inhibit the expression of their downstream target genes. IDRs and proline specific residues of EBNA2 and EBNALP contribute to the formation phase separation droplet 201. In addition, EBNA2 can regulate cell alternative splicing events by interacting with components of the splicing mechanism. When the phase separation of EBNA2 is destroyed with 1,6 hexanediol, its ability to regulate splicing can be effectively inhibited 202. These findings suggest that EBNA2 regulates the transcription of downstream genes as a transcription factor and regulates the splicing of downstream genes as a splicing factor both depend on its phase separation properties. Since 1,6 hexanediol has a wide range of properties of destroying phase separation condensates, further studies are needed to specifically destroy the phase separation ability of EBNA2 by mutating proline residues of EBNA2. These findings provide a basis for understanding the mechanism of phase separation in EBV-host cell interaction and involved in controlling target gene expression. It also provides a new idea for the treatment of EBV-related cancers.

### LLPS in KSHV

KSHV belongs to γ-Herpesvirus, a common DNA virus, has been considered to be related to a variety of human malignant tumors, including Kaposi's sarcoma and lymphoma 203. Immunohistochemistry showed that latency-associated nuclear antigen (LANA) was expressed in almost all KSHV-related tumors, which showed that LANA could be used for diagnosis in KSHV-infected tumors 204. LANA can recruit host machinery into the viral genome, thus playing a key role in the latency of KSHV 205. Destroying the phase separation of LANA can change the chromosome conformation of KSHV. During KSHV lytic reactivation to form LANA-related replication compartments, LANA nuclear bodies undergo major morphological transitions. These findings suggest the LANA nucleosome is a dynamic molecular condensates dependent on phase separation, and undergo morphological changes corresponding to different modes of virus replication 206.

### LLPS in HPV

Human papillomavirus (HPV) may cause HPV-related tumor, which is mainly transmitted through sexual behavior 207. About 5% of cancers worldwide are associated with HPV infection, including all cervical cancers and an increasing number of oropharyngeal cancers 208. The types 16 and 18 HPV are the most common causes of HPV-associated tumors, with about 70% of precancerous cervical lesions and cervical cancer 207. The genome of HPV encodes for six nonstructural viral proteins (E1, E2, E4, E5, E6, and E7) from the early region of the viral genome. E1 and E2 proteins are mainly involved in viral DNA replication and early transcriptional regulation. As viral oncogenes, E5, E6 and E7 can promote cell transformation and immortalization 209. HPV infection can induce the formation of HPV E1/E2 foci or replication foci, which contain the main regulatory factors of viral helicase E1 and E2, and DDR protein. These foci have been reported to form by means of phase separation 206. In addition, super-enhancers condensate has been reported to be regulated by phase separation 137, formation of super-enhancers condensates have been postulated as a new way of HPV-16 integration 210, highlighting the potential role of phase separation in viral tumorigenesis 211.

## LLPS in cancer therapy

With the increasing understanding of the role of LLPS in many biological processes, it is gradually recognized that LLPS can regulate some tumor-associated proteins and their downstream and upstream signal pathways, so as to achieve tumor targeted therapy. Recently, Klein found that antitumor drugs are enriched in specific protein droplets in vitro, which occurs via physicochemical characteristic independent of drug targets. This phenomenon has also been found in cancer cells. The distribution of drugs in tumor cells can affect the activity of drugs. Moreover, changing the properties of phase separation condensate affects the concentrations and activity of the drug. These studies showed that the selective distribution and concentration of drugs in the phase separation condensates are helpful to the pharmacodynamics of drugs, and a further understanding of this phenomenon may be helpful to the research of cancer treatment in the future^212^, ^213^.

Anti-PD-L1/PD-1 therapy has shown good clinical effects in a large number of types of tumors, but drug resistance has also appeared in solid tumors, including primary, adaptive and acquired problems 214-218. Recently, Yu, et al. reported that YAP undergo nuclear translocation and LLPS after IFN promoted anti-PD-1 treatment of cancer cells. YAP interacts with histone acetyltransferase EP300, transcription factor TEAD4 and mediator1 to form phase separation transcriptional condensates, so as to promote target gene transcription. Destroying the LLPS ability of YAP can inhibit cancer cells growth, enhance immune response and make tumor cells sensitive to anti-PD-1 therapy. The prognosis of tumor patients was negatively correlated with YAP activity. These results indicate that YAP regulates the IFN-γ pro-tumor effect via its LLPS ability and shows that YAP can be used as a predictive biomarker and target of anti-PD-1 combination therapy 215. These studies suggest that the development of drugs regulating LLPS may be a potential way to treat cancer with abnormal condensates and protein aggregates.

## Conclusions

Up to now, more and more cancer-related proteins with phase separation function have been discovered and identified, and their regulation through phase separation may affect their cellular domain, function and the life processes of cancer cells. In this review, we have presented recent findings that phase separation regulates tumor-related signaling pathways and reviewed some tumor virus-associated proteins to regulate the development of virus-associated tumors via phase separation; we have also discussed some possible strategies for treating tumors by targeted phase separation. In recent years, the study of phase separation has provided valuable opportunities for in-depth understanding of the pathophysiological process of organisms and the occurrence and development mechanism of various diseases. However, phase separation is a young biological research direction, and there are still many problems to be solved on how to accurately control phase separation. Currently, studies on the role of phase separation in the regulation of cancer mainly focus on the role of condensates formed by phase separation in cancer cells. However, the specific mechanism of the dynamic characteristics of phase separation in the occurrence and development of tumors is still lacking. Therefore, the relationship between phase separation and cancer still needs further exploration. 1,6 hexanediol is the most important chemical to destroy condensates, which can be used to explore the biological function of condensates. However, there is no specificity for 1.6 hexanediol to destroy the condensates in cells, so more specific methods to destroy phase separation need to be urgently explored. For example, gene editing techniques can investigate the function of protein condensates by specifically disrupting the protein phase separation ability by changing the amino acids necessary for phase separation. At present, many studies have verified whether proteins can undergo phase separation by purifying proteins in vitro. However, due to the complexity of the intracellular environment and molecular regulation mechanism, the simulated conditions in vitro cannot completely replace the intracellular environment. Fortunately, with the progress of optogenetics, more and more phase separation studies have been applied to optogenetics. Compared with previous studies on protein and nucleic acid phase separation mainly by means of in vitro reconstruction, optogenetics can be used to dynamically observe the interaction of proteins in living cells, thus realizing the spatiotemporal control of phase separation in living cells. At present, the main methods to study the phase separation of macromolecules are to label them with fluorescence, and then use ordinary optical microscope to detect whether they form droplets or carry out fluorescent recovery after photobleaching experiments. However, ordinary microscope can only observe the state of fluorescent labeled protein or nucleic acid at a fixed time point, and cannot know the dynamic changes of phase separation condensate. Therefore, monomolecular magnetic resonance, atomic force microscopy, cryo-electron microscopy and other techniques should be more used to study the internal structure and dynamic characteristics of biological macromolecular condensates.

## Funding

This work was supported in part by grants from the following sources: the National Natural Science Foundation of China (82173142, 81972636, 81872281, 81772842), the Natural Science Foundation of Hunan Province (2020JJ5336, 2019JJ40175, 2019JJ40183), the Research Project of Health Commission of Hunan Province (202203034978, 202109031837, 20201020, C2019066), Hunan Provincial Science and Technology Department (2020TP1018), Changsha Science and Technology Project (kh2201054), Ascend Foundation of National cancer center (NCC201909B06, NCC2018b68), and supported by Hunan Cancer Hospital Climb Plan (ZX2020001-3, YF2020002).

## Author contributions

Qiu Peng, Shiming Tan, Longzheng Xia, Nayiyuan Wu, Linda Oyang, Yanyan Tang, Min Su, Xia Luo, Ying Wang, Xiaowu Sheng collected the related paper and drafted the manuscript. Yujuan Zhou and Qianjin Liao revised and finalized the manuscript. All authors read and approved the final manuscript.

## Figures and Tables

**Table 1:** Membraneless organelles formed by phase separation

Name	Location	Functions	References
Nucleoli	Nucleus	The site of ribosomal RNA (rRNA) production and ribosome subunit assembly	219
P-bodies	Cytoplasm	Associated with translation repression and 5'-to-3'mRNA decay	220
Cajal bodies	Nucleus	Involved in the formation of ribonucleoproteins including small nuclear RNPs	221
PML bodies	Nucleus	Involved in a wide variety of biological processes ranging from senescence to viral infections or stemness	222
Stress granules	Cytoplasm	Play an important in the stress response and may contribute to some degenerative diseases	223
U-bodies	Cytoplasm	Involved in mRNA decay and translational repression	224
Paraspeckles	Nucleus	Mediate the nuclear retention of some A-to-I hyper-edited mRNAs, gene transcription, RNA splicing, and RNA stability	225
GW/P bodies	Cytoplasm	Translational repressors of mRNA through Ago2-mediated RNA silencing	226
Polycomb bodies	Nucleus	Mediate down-regulation of target genes	227
Nuclear speckle	Nucleus	Inhibition of mRNA splicing	228

**Table 2:** Phase separation related databases

Name	URL	Functions
IUPred	https://iupred2a.elte.hu/plot	Prediction of disordered protein regions
PLAAC	http://plaac.wi.mit.edu/	Prediction of prion-like region
PONDR	http://www.pondr.com	Predictor of natural disordered regions
MobiDB	https://mobidb.org	Provides information about intrinsically disordered regions and related features
CIDER	http://pappulab.wustl.edu/CIDER/	Calculation of many different parameters associated with disordered protein sequences
ZipperDB	https://services.mbi.ucla.edu/zipperdb/	Predictions of fibril-forming segments within protein
D2P2	http://d2p2.pro/	Database of disordered protein predictions
Metadisorder	http://iimcb.genesilico.pl/metadisorder/	Prediction of protein disorder
Expasy	https://web.expasy.org/compute_pi/	Computation of the theoretical pI (isoelectric point) and Mw (molecular weight)
AMYCO	http://bioinf.uab.es/amycov04/	Evaluation of mutation impact on prion-like proteins aggregation propensity
RPS	http://rps.renlab.org/#/Home	A comprehensive database of RNAs involved in liquid-liquid phase separation
RNAPhaSep	http://www.rnaphasep.cn/#/Home	A resource of RNAs undergoing phase separation
LLPSDB	http://biocomp.ucas.ac.cn/llpsdb/home.aspx	A database of proteins undergoing liquid-liquid phase separation in vitro

## Abbreviations

LLPS: liquid-liquid phase separation
IDRs: intrinsically disordered regions
P-bodies: processing bodies
SGs: stress granules
LCRs: low-complexity regions
RBPs: RNA-binding proteins
PLDs: prion-like domains
PRMs: proline-rich motifs
N-WASP: neural Wiskott-Aldrich syndrome protein
SH3: SRC homology 3
FUS: fused in sarcoma
TDP-43: transactivation response element DNA-binding protein 43
EWSR1: Ewing sarcoma breakpoint region 1
TAF15: TATA-binding protein-associated factor 15
HnRNPA: heterogeneous nuclear ribo-nucleoproteins A
LncRNAs: long noncoding RNAs
m6A: RNA N6-methyladenosine
eRNA: enhancer RNAs
ALS: amyotrophic lateral sclerosis
FTD: frontotemporal dementia
53BP1: p53-binding protein 1
PKA: protein kinase A
SMN: survival motor neuron
ATG13: autophagy related gene 13
PAS: pre-autophagosomal structure
cGAS: cyclic GMP-AMP
TCR: T cell receptor
BCR: B cell receptor
LAT: linker for activation of T cells
